# Questionnaire-assessed genotypes and associations with symptoms in primary ciliary dyskinesia

**DOI:** 10.1183/23120541.00288-2024

**Published:** 2024-10-28

**Authors:** Eva S.L. Pedersen, Myrofora Goutaki, Leonie D. Schreck, Bernhard Rindlisbacher, Lucy Dixon, Jane S. Lucas, Claudia E. Kuehni, the COVID-PCD patient advisory group

**Affiliations:** 1 Institute of Social and Preventive Medicine, University of Bern, Bern, Switzerland; 2 Department of Clinical Research, University of Bern, Bern, Switzerland; 3 Division of Paediatric Respiratory Medicine and Allergology, Department of Paediatrics, Inselspital, University Hospital, University of Bern, Bern, Switzerland; 4 Graduate School for Health Sciences, University of Bern, Bern, Switzerland; 5 Selbsthilfegruppe Primäre Ciliäre Dyskinesie, Steffisburg, Switzerland; 6 PCD Support UK, Buckingham, UK; 7 Primary Ciliary Dyskinesia Centre, NIHR Biomedical Research Centre, University Hospital Southampton NHS Foundation Trust, Southampton, UK; 8 University of Southampton Faculty of Medicine, School of Clinical and Experimental Medicine, Southampton, UK; 9 For a list of the COVID-PCD patient advisory group members and their affiliations, see the Acknowledgements section

## Abstract

Knowledge about genotype–phenotype associations is crucial for understanding the clinical variability of primary ciliary dyskinesia (PCD) [1]. More than 50 known disease-causing genes lead to different ciliary structural defects resulting in differing phenotypical characteristics [2]. For example, laterality defects are not associated with mutations in genes related with the central pair apparatus, nexin link or radial spoke complex (*e.g. HYDIN* and *RSPH9*), while they are seen in approximately 50% of individuals with mutations in most other genes [3, 4].

## Shareable abstract

**Collecting genetic information by questionnaire from people with PCD is feasible. This study confirmed typical differences in laterality defects and age at diagnosis between genotypes but found that symptoms were equally common across genotypes.** https://bit.ly/4bvKW4R

*To the Editor:*

Knowledge about genotype–phenotype associations is crucial for understanding the clinical variability of primary ciliary dyskinesia (PCD) [1]. More than 50 known disease-causing genes lead to different ciliary structural defects resulting in differing phenotypical characteristics [2]. For example, laterality defects are not associated with mutations in genes related with the central pair apparatus, nexin link or radial spoke complex (*e.g. HYDIN* and *RSPH9*), while they are seen in approximately 50% of individuals with mutations in most other genes [3, 4]. Similarly, congenital heart disease is less frequent in patients with these genes as congenital heart disease is associated with laterality defects [5, 6]. Several studies investigated associations of lung function with genotype and reported steeper decline in forced expiratory volume in 1 s for people with mutations affecting microtubular stabilisation (*e.g. CCDC39* and *CCDC40*) compared to mutations in other genes [7–9].

Few studies have investigated differences in symptoms between genotypes, with inconsistent results. Data from 118 children from North America showed little difference in prevalence of chronic cough, chronic nasal congestion, and neonatal respiratory distress between groups of different genotypes [10]. Data from 396 individuals from the UK, France and the Netherlands in contrast showed lower prevalence of neonatal respiratory distress among patients with causative genes affecting the dynein structure compared to other genotypes [9].

Large studies including both detailed patient-reported symptom data and genetic data from medical records can be difficult to design. We therefore first studied how feasible it is to collect information about causative genes directly from people with PCD through questionnaires, and second investigated associations between clinical characteristics, symptoms and genotype.

We used data from the anonymous international participatory cohort COVID-PCD, a study set up in 2020 to follow people with PCD during the COVID-19 pandemic and beyond (clinicaltrials.gov: NCT04602481) [11, 12]. The study is online and includes people of any age worldwide. Study participants registered through the study website (www.covid19pcd.ispm.ch) and provided online consent (ethical approval from the Cantonal Ethics Committee of Bern, Switzerland; study ID: 2020-00830). The baseline questionnaire asked about diagnostic tests (including genetic analysis), whether participants knew the results, and if yes, participants could select their causative gene through a list of the 53 PCD-causing genes known as of May 2020 [13]. We also asked about age at diagnosis, laterality defects, congenital heart disease, and detailed questions about symptoms during the past 3 months.

We grouped reported genes into categories based on associated defects: 1) dynein structure (DS), including *DNAH5*, *DNAH11*, *DNAI1*, *DNAI2*, *ODAD2*, *DNAH9*, *ODAD4, ODAD1 (CCDC114)*; 2) dynein assembly (DA), including *CCDC103*, *DNAAF4*, *LRRC6 (DNAAF11)*, *DNAAF3*, *SPAG1 (DNAAF13)*, *ZYMND10 (DNAAF7)*, *DNAAF5*, *CFAP300 (DNAAF17)*; 3) microtubular stabilisation/nexin–dynein regulatory complex (N-DRC), including *CCDC39*, *CCDC40*, *CCDC65 (DRC2)*, *DRC1*; 4) radial spoke and central complex (RS-CC), including *RSPH4A*, *RSPH1*, *HYDIN*, *RSPH9*, *RSPH3*; and 5) other function (*RPGR*, *CCNO*, *MCIDAS*) [9]. We also compared differences for single causative genes reported by more than five people.

Our study included 759 people with suspected or confirmed PCD with median age of 28 years (interquartile range 13–44, range 1–86), 452 (60%) were females. Participants came from 49 countries; most from the USA (n=132), England (n=128), Germany (n=111), Italy (n=52), and Switzerland (n=50).

In total, 444 (58%) reported genetic testing; 52 (11%) reported no mutation found; 103 (24%) either did not know or awaited results; and 289 (65%) reported a gene was identified. Among these, 229 (79%) participants knew and reported the causative gene. We excluded 23 participants with heterozygous mutations in different genes, leaving 206 in our analysis. The most common genes were *DNAH5* (n=71; 34%), *DNAH11* (n=27; 13%), *CCDC40* (n=21; 10%), *DNAI1* (n=18; 9%), *CCDC39* (n=13; 6%) and *RSPH1* (n=8; 4%).

The DS group was the largest (n=127) followed by the N-DRC group (n=38), DA group (n=21), and RS-CC group (n=20) (table 1). Current age and sex were similar across groups, but median age at diagnosis was markedly higher in the RS-CC group at 11 years, compared to 4–7 years in the other groups (p=0.035). Laterality defects were reported by one person (5%) in RS-CC group, compared with 37–60% in other groups (p=0.001). Overall, symptoms were frequently reported by participants in all four groups with little difference between groups (table 1). Prevalence of cough (reported either daily, often or sometimes during the past 3 months) ranged from 84% to 92% (p=0.474); ear pain between 33% and 43% (p=0.772); and shortness of breath between 45% and 62% (p=0.589) across genotype groups. The biggest difference was seen for chronic nose symptoms which was reported by 62% in the DA group and 86% in the DS group (p=0.064).

**TABLE 1:** Clinical characteristics and symptoms by genotype groups including dynein structure (DS), dynein assembly (DA), nexin–dynein regulatory complex (N-DRC), and radial spoke and central complex (RS-CC) (n=206), and by single genes including *DNAH5*, *DNAH11*, *CCDC40*, *DNAI1*, *CCDC39* and *RSPH1* (n=158)

	Gene groups					Single genes						
	DS^#^	DA^¶^	N-DRC^+^	RS-CC^§^	p-value^ƒ^	*DNAH5*	*DNAH11*	*CCDC40*	*DNAI1*	*CCDC39*	*RSPH1*	p-value^ƒ^
	n=127	n=21	n=38	n=20		n=71	n=27	n=21	n=18	n=13	n=8	
**Age, years**	21 (10–42)	19 (7–37)	19 (9–37)	29 (7–43)	0.776	23 (10–41)	25 (12–45)	22 (11–41)	16 (8–42)	11 (5–23)	23 (7–47)	0.379
**Age at diagnosis, years**	7 (2–18)	4 (1–6)	6 (0–9)	11 (12–38)	0.035	6 (2–12)	10 (6–31)	6 (2–9)	6 (0–22)	1 (0–3)	12 (4–30)	0.019
**Sex (female)**	73 (57)	11 (52)	23 (61)	13 (65)	0.844	43 (61)	16 (59)	15 (71)	9 (50)	6 (46)	5 (63)	0.709
**Clinical characteristics**												
Laterality defect	59 (48)	13 (62)	14 (37)	1 (5)	0.001	33 (46)	13 (48)	7 (33)	7 (44)	7 (54)	1 (13)	0.408
Congenital heart disease	9 (7)	3 (14)	8 (21)	2 (10)	0.098	4 (6)	3 (11)	4 (19)	1 (6)	4 (31)	0 (0)	0.055
Bronchiectasis	80 (66)	11 (61)	25 (68)	13 (68)	0.946	49 (72)	15 (58)	14 (70)	10 (59)	7 (54)	6 (75)	0.608
Hospitalised past year	25 (20)	2 (10)	12 (32)	2 (10)	0.118	14 (20)	7 (27)	9 (43)	4 (22)	2 (15)	1 (13)	0.294
**Reported symptoms^##^**												
Upper respiratory												
Chronic nose symptoms	109 (86)	13 (62)	31 (82)	17 (85)	0.064	59 (83)	25 (93)	18 (86)	15 (83)	11 (85)	7 (88)	0.910
Headache when bending down	15 (12)	2 (10)	3 (8)	4 (20)	0.578	9 (13)	2 (7)	1 (5)	2 (11)	1 (8)	1 (13)	0.908
Ear pain	55 (43)	7 (33)	15 (39)	7 (35)	0.772	31 (44)	14 (52)	7 (33)	6 (33)	7 (54)	3 (38)	0.687
Ear discharge	33 (26)	4 (19)	11 (29)	2 (10)	0.364	21 (30)	5 (19)	5 (24)	3 (17)	4 (31)	0 (0)	0.412
Hearing problems	61 (50)	11 (55)	24 (67)	8 (42)	0.254	35 (51)	13 (52)	14 (70)	6 (33)	8 (67)	3 (38)	0.234
Lower respiratory												
Cough	116 (92)	19 (90)	32 (84)	17 (85)	0.474	66 (93)	24 (92)	18 (86)	16 (89)	12 (92)	7 (88)	0.929
Sputum	109 (88)	20 (95)	35 (92)	19 (95)	0.301	62 (90)	24 (89)	20 (95)	14 (82)	12 (92)	8 (100)	0.729
Wheeze	60 (49)	9 (43)	23 (62)	11 (58)	0.394	39 (57)	12 (48)	12 (60)	5 (28)	9 (69)	3 (43)	0.204
Shortness of breath	64 (52)	9 (45)	23 (62)	10 (50)	0.589	38 (54)	15 (58)	14 (67)	6 (33)	8 (67)	4 (50)	0.370
Generally feeling unwell	54 (43)	12 (57)	18 (50)	10 (53)	0.581	23 (33)	14 (52)	12 (60)	9 (50)	5 (42)	2 (25)	0.201

Data are presented as n (%) or median (interquartile range). ^#^: *DNAH5* (n=71), *DNAH11* (n=27), *DNAI1* (n=18), *DNAI2* (n=3), *ODAD2* (n=2), *DNAL1* (n=1), *DNAH9* (n=2), *ODAD1 (CCDC114)* (n=4); ^¶^: *CCDC103* (n=1), *DNAAF4* (n=3), *LRRC6* (n=3), *DNAAF3* (n=1), *SPAG1* (n=4), *ZYMND10* (n=3), *DNAAF5* (n=2), *CFAP300* (n=1), *DNAAF1* (n=1); ^+^: *CCDC39* (n=13), *CCDC40* (n=21), *CCDC65* (n=4); ^§^: *RSPH4A* (n=2), *RSPH1* (n=8), *HYDIN* (n=5), *RSPH9* (n=5); ^ƒ^: from Kruskal–Wallis or chi-squared; ^##^: symptoms reported either daily, often, or sometimes during the past 3 months.

Our analysis comparing single genes similarly showed difference in age at diagnosis with lowest age in participants with *CCDC39* (1 year) and highest age of diagnosis in participants with *RSPH1* (12 years) (p=0.019). Laterality defect was reported in one person with *RSPH1* (13%) and between 33% and 54% in all other groups (p=0.408). Congenital heart defect ranged from 0% in *RSPH1* to 31% in *CCDC39* (p=0.055). Again, symptoms were frequent across single genes.

This study using data contributed directly by people with PCD demonstrates that it is feasible to collect genetic information at the gene level by questionnaire. Among study participants who reported genetic testing, two thirds reported that a causative PCD gene had been identified, and among these most participants (four out of five) were able to report the specific causative gene. In line with predicted causative gene prevalence worldwide, we found that *DNAH5*, *DNAH11* and *CCDC40* were most frequent [14]. Our findings reinforce previously shown genotypic differences. Laterality defect was not associated with the RS-CC group, in line with other studies [4, 15]. This may explain that age at diagnosis was highest for the RS-CC group, as people without laterality defects are often diagnosed later [16]. We found a higher prevalence of congenital heart disease for *CCDC39* and *CCDC40* than previously reported for PCD [4, 5, 9]; there is, however, no mechanism to explain this finding. We found that study participants reported frequent symptoms independent of genotype or single genes, suggesting that from a patient perspective, no phenotype without frequent symptoms exists, although some genotypes, such as *DNAH11*, are considered milder and associated with preserved lung function [9].

The major strengths of our study include the sample size, with 206 people, and the combination of standardised patient-reported symptoms and genetics data, which allowed investigating novel genotype–phenotype associations. Our study is limited by our use of self-reported genetic data, which cannot be validated through linkage with clinical records from our anonymous study design. Some misclassification of genotype might have occurred, as for example one person with *RSPH1* reported laterality defect; *RSPH1* is a gene not present in the embryonic node, which cannot cause laterality defect. Without detailed clinical data on the mutation level, which patients usually do not have, we cannot be certain that all reported variants were pathogenic and that no single heterozygous variants or variants of unknown significance were reported as pathogenic. However, our data led to similar associations as previous studies which had used genetic information from clinics, suggesting that patient-reported genetic information can be reliable for epidemiological studies. Our results confirmed known differences in laterality defects and congenital heart disease between genotypes and showed frequent upper and lower respiratory symptoms in all groups, regardless of reported gene.
